# Developments in tandem ion mobility mass spectrometry

**DOI:** 10.1042/BST20190788

**Published:** 2020-12-18

**Authors:** Charles Eldrid, Konstantinos Thalassinos

**Affiliations:** 1 Institute of Structural and Molecular Biology, UCL, Gower St, London WC1E 6BT, U.K.; 2 Institute of Structural and Molecular Biology, Birkbeck University, Malet Place, London WC1E 7HX, U.K.

**Keywords:** ion mobility, mass spectrometry, protein structure, tandem ion mobility

## Abstract

Ion Mobility (IM) coupled to mass spectrometry (MS) is a useful tool for separating species of interest out of small quantities of heterogenous mixtures via a combination of *m/z* and molecular shape. While tandem MS instruments are common, instruments which employ tandem IM are less so with the first commercial IM–MS instrument capable of multiple IM selection rounds being released in 2019. Here we explore the history of tandem IM instruments, recent developments, the applications to biological systems and expected future directions.

## Introduction

Mass spectrometry (MS) was once the purview of select chemists and physicists, but is now one of the most widely used analytical methods used by researchers in many other scientific disciplines. It is used to analyse a range of analytes from biopharmaceuticals [1, 2] to explosives [3], and used in fields ranging from life-sciences to ecology [4] and petroleomics [5]. MS has also been an integral part of many space missions [6].

A major key to the success of MS has been the ability to perform tandem MS experiments whereby ions of interest are selected during the first MS analysis stage, dissociated (using a number of different approaches such as collision-induced dissociation [7, 8], electron capture dissociation [9] and infrared photodissociation [10, 11]), with the fragments analysed during the second stage of MS. Tandem MS therefore allows the detailed structural characterisation of ions and has been an integral part of proteomics and other omics fields.

Ion mobility (IM) and, in particular, ion mobility coupled to mass spectrometry (IM–MS), appears to be heading in the same direction. An increasing number of practitioners are using it as part of their analytical toolbox, especially since the development of commercial instrumentation in 2006 [12]. IM functions by separating molecules according to their *m/z* and reduced mobility *K* in a neutral buffer gas. IM–MS can be used to calculate the momentum transfer collision cross section (CCS) [13] of an analyte, a descriptor closely related to its three-dimensional structure. Used in conjunction with theoretically calculated CCS values, structures for species of interest can be assigned to the experimental data. IM–MS also provides an extra level of analytical separation which can be used to distinguish isobaric species, something not possible using mass spectrometry alone.

Analysis of biological molecules using IM–MS has provided new insights into systems that are not easily studied by other biophysical and structural approaches. The resulting mobility spectra however can be very complex, especially for proteins, and increases in the IM resolving power alone cannot resolve the multiple overlapping conformational families present in the spectra [14, 15]. In an analogous approach to tandem MS, tandem IM coupled to MS can overcome the limitations of single IM analyses, especially when ions are activated between the two IM rounds.

In this review, we introduce the fundamentals of ion mobility and discuss different ion mobility technologies. We then outline the history of the development of tandem ion mobility–mass spectrometry and discuss expected future advances especially with regards to the analysis of biological molecules.

## Ion mobility

Ion mobility (IM), or ion mobility spectrometry (IMS), is rooted in the same physics which birthed mass-spectrometry over 100 hundred years ago and predates MS [16]. In the archetypal drift-tube ion mobility spectrometry (DTIMS) setup, ions moving through a neutral gas under a constant electric field experience a reduced mobility, as collisions occur between the ion and the buffer gas. Ions of the same mass and charge, but different three-dimensional structures, will have different reduced mobilities through the buffer gas: more extended structures will undergo a greater number of collisions and so will take longer to traverse the drift-region than a compact structure. This reduced mobility *K* can be used to calculate a collision cross-section (CCS) according to the Mason–Schamp equation [17]:

$$\Omega =\frac{3ez}{16N}{\left(\frac{2\pi }{\mu k_{B}T}\right)}^{\frac{1}{2}}\;\frac{1}{K}$$

where Ω is CCS, *e* is the elementary charge, *z* is the charge state of the analyte ion, *N* is the density of the drift-gas, µ the reduced mass of the collision ion, *k_B_* the Boltzmann constant at temperature *T*.

The CCS can be used as an effective empirical factor for a variety of biological systems. For instance, in the field of glycomics, it is inherently difficult to assign structures due to the wide variety of isomerism which occurs, however CCS has been used to differentiate between different glycan structures with differential branching [18]. In small molecule analysis CCS can be used as an additional identification factor [19]. IM has also been used in conjunction with native MS to study the dynamics of protein structures [20], fibril formation in amyloid-β [21], and investigate the structural dynamics of disordered proteins — a notoriously difficult system to work with [22–24].

Since the development of DTIMS, there have been numerous other IM methods developed, such as ones where the CCS can be measured directly from reduced mobility: differential mobility analysis (DMA) [25], overtone mobility spectrometry (OMS) [26] and transversal modulation IMS (TMIMS) [27], or where it can be obtained through the use of calibrants [28]: travelling wave IMS (TWIMS) [29] and trapped IMS (TIMS) [30]. IM methods have been discussed in detail elsewhere [13], however we will briefly revise here how different IM methods function.

As previously described, DTIMS functions by applying a constant electric field across the drift-region and therefore the velocity of analyte ions can be described by the Mason–Schamp equation. TWIMS functions by inducing electric fields in consecutive plates within stacked ring ion guides (SRIGs) to create an electrical wave upon which ions ‘*surf’* [12, 29, 31]. Both DTIMS and TWIMS are described as *time-dispersive* as their mobility is defined as the time it takes for each ion to traverse the drift-region with a static gas. Both DMA [25] and field asymmetric waveform IMS (FAIMS) [32, 33] utilise gas which flows perpendicular to a modulated electric field, ions which have a certain mobility are able to traverse the drift-region and pass through an aperture. The difference between DMA and FAIMS is that DMA applies a constant electric field, which is increased or decreased incrementally to obtain an IM spectrum. In comparison FAIMS applies pulses of opposing electrical fields, and so CCS cannot be calculated, unlike with DMA. These are described as *spatially dispersive*. TIMS utilises an electric field which opposes a gas flow to trap analyte ions. The gating field can then be dropped incrementally and the ions traverse the drift-region. The ions with the largest CCS experience the greatest force through the drift-region [30, 34]. Due to the elution of the ions from the trap being controlled by their movement against the electric field, TIMS can be described as *field-dispersive*. Recently a new U-shaped mobility analyser (UMA) was developed which features flow and counter-flow separation, comparable to a mixture of DMA and TIMS separation [35].

An important consideration of IM–MS instruments is the resolving power: the ability for an instrument to separate two closely eluting species. Simplistically, resolving power R can be described as:

$$\text{R}=\frac{t_{d}}{\text{w}}$$

Where *t*_d_ is drift time at the peak top and w is the full-width half maximum of the peak [36]. It has also been described in terms of *t*_d_/Δ*t*_d_ or CCS/ΔCCS. The definitions where R is based on *t*_d_ are applicable to IM techniques where *t*_d_ scales proportionally with CCS, which is not the case for techniques such as TWIMS and TIMS. Therefore, the definition CCS/ΔCCS should be considered the most accurate definition of R. Resolving power calculations can be optimised by using more experimental factors, however they are IM method dependent. For instance in DTIMS the R can be increased by increasing the electric field strength, or in TWIMS by altering the velocity of the wave [37].

**Figure 1.**
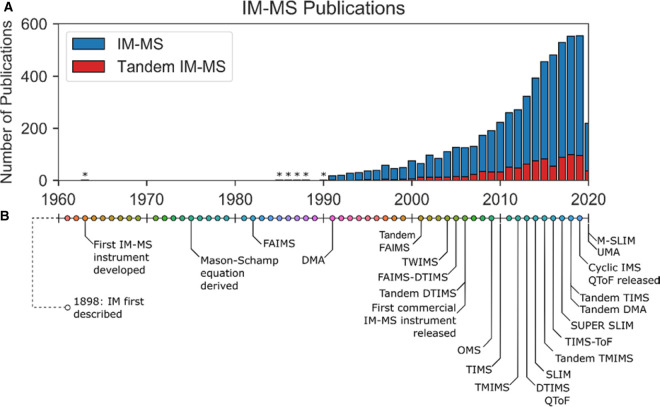
History and Development of IM-MS and Tandem IM. (**A**) History of IM–MS and Tandem-IM publications, from searches on Web of Science for topics: IM–MS; Tandem-IM. Astrices represent where there were fewer than 10 publications per year (**B**) Timeline of advances of IM, IM–MS and Tandem-IM, with the dating from 1960–2020. References, in chronological order: [16, 25, 27, 29, 30, 32, 33, 35, 38–49].

Ion mobility–mass spectrometry (IM–MS) has been a rapidly expanding field since the 1990s, when publications became more numerous, and has been increasing at an exponential rate (Figure 1A). Multiple mass spectrometry companies have released their own IM–MS instruments, such as the first commercial IM–MS instrument the Synapt (Waters Corp, UK), the FAIMS Quantum (Thermo), 6560 IM QToF (Agilent), ABSciex Selexion (Sciex), IMS-TOF (Tofwerk), and the TIMS-ToF (Bruker).

## Development of tandem IM

Another field which has been steadily growing is tandem IM (Figure 1A), whereby multiple rounds of mobility selection can take place, analogous to tandem MS where successive rounds of mass analysis can occur. Tandem MS instruments can provide useful parallels which can be applied to the description of tandem IM instruments, highlighted in the comparison between triple quadrupole and quadrupole ion trap MS instruments [50]. Triple quadrupoles are *tandem-in-space* where ion selection and activation occur in separate regions of the instrument. Quadrupole ion traps, where ions are accumulated in a trap and then analysis and fragmentation occur in the same space, are *tandem-in-time.* Both tandem-in-space and tandem-in-time IM–IM instruments have been developed. It should be noted that in this review we will only be discussing tandem IM coupled to mass-spectrometry; other types of tandem IM, such as the stand-alone DMA–DMA [51], FAIMS–DMA [52] and the DMA–DTIMS [38] used for aerosol analysis, will not be discussed.

The first tandem IM instrument was developed in 2001 by the National Measurements Standards of Canda and was a multi-FAIMS separation system, where three FAIMS cells were arranged perpendicularly. The first and the third FAIMS cells acted as separation cells, while the second acted as a trapping cell [53]. The instrument was developed in order to increase the limits of detection of small analyte ions through selection and accumulation of an ion species, which could then be introduced as a single ion cloud.

The applications of FAIMS in tandem IM was expanded to be coupled to a DTIMS cell in 2005 [39]. The FAIMS cell was used to scan through different mobilities using a shifting compensation voltage, which allowed greater sensitivity in the DTIMS cell. This was used initially for increasing the limits of detection for complex peptide mixtures, and was used in 2006 to study the protein conformations of ubiquitin, making it the first use of tandem IM for native proteins [39, 54]. The DTIMS separation post-FAIMS was able to show a greater number of measurable conformers than had been seen previously [54–58].

**Figure 2.**
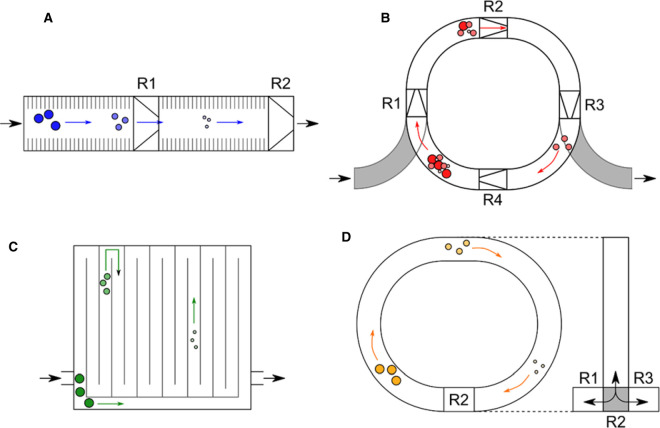
Example geometries of select tandem-IM instruments. (**A**) tandem DTIMS presented by Koeniger et al. [40] where ion packets can be transmitted, selected or activated in regions R1 and R2 (**B**) ion cyclotron presented by Merenbloom et al. [61] where ion packets of particular mobilities are selected for in regions R1–R4 (**C**) SUPER-SLIM presented by Deng et al. [43] where ion packets can be pushed to either travel through or bypass a serpentine IM track **D)** cyclic IM-QToF presented by Giles et al. [47] where ion packets can be stored and activated in regions R1 and R3, and introduced to the cyclic IM guide and activated in region R2.

At a similar time in 2006, the Clemmer group developed a tandem DTIMS instrument, comprising separate drift-tubes flanked by funnel gating systems with a total R ∼ 500 (∼50 for the first DTIMS section and ∼10 for the second) (Figure 2A) [40, 59]. This geometry allowed a variety of experiments to be performed, depending on how the gating systems (Figure 2A, R1 and R2) were used. R1 or R2 could allow full ion transmission, which resulted in greater IM resolution for all species; as gating devices allowing only a single species to be transmitted for mobility separation; or as an ion activation cell, allowing fragmentation pre- or post-mobility. The same year a three drift-cell IM–MS instrument, using the same geometry of drift-cell flanked by ion funnels, was developed by the same group [41]. These geometries could be used flexibly and the first experiments were initially used to increase peptide fragmentation coverage by using the mobility selection and activation [40, 41, 60].

These linear IM–IM instruments were also used for native MS analysis of well-characterised proteins such as ubiquitin [14]. By taking variable mobility selections and activating them it was possible to directly track stable and unstable precursor conformations and their transitional and final states. This sort of methodology is of particular interest to those who study protein unfolding or misfolding, as the majority of other structural biology techniques are only able to measure ensemble states. Indeed these systems were used to develop a new method of ion mobility separation: overtone mobility spectrometry (OMS) [62]. One observation of utilising the funnel regions present in the linear tandem DTIMS instrument for mobility selection was an increase in resolution. Ions which have the same resonance as the frequency of the gating funnel have a mobility resolution which is higher than that of the resolving power given by the frequency, hence the designation *overtone* [26, 62–64].

OMS was limited by the number of funnels which an ion packet could pass through, so efforts were made to create an IM–MS instrument with a circular drift-region [61]. This has clear advantages over linear instruments as the total drift distance can be incrementally increased. The ion cyclotron described in 2009 had funnels connecting curved DTIMS regions and allowed refocusing of ions to prevent loss of signal (Figure 2B) [61]. The electric fields across these regions is applied at a selected frequency, and the field is switched between each segment and funnel. The function of this is that only ions with mobilities resonant to the frequency of field application are able to proceed to the following segment, whilst others are removed. Simplistically, ‘*trimming*’ of the ion packet occurs by the gating funnels to allow selection of a single mobility species and increasing the mobility resolution of that species [61]. While the trimming of the ion packets increased the mobility resolution up to R > 1000, possibly artificially, the sensitivity of the system is reduced due to the continual loss of ions due to non-resonance with the drift-field frequency. The sensitivity of the ion cyclotron can be increased by using a different trapping mechanism allowing multiple ion packets to be introduced prior to mobility separation [65]. Another limitation of OMS and the cyclotron in particular is that the only ions which have a stable conformation can be transmitted [66] and early work by the Clemmer group specifically showed that the conformations of proteins in the gas phase can decay over time-scale of milliseconds under certain conditions [58, 67, 68].

## A decade of expansion

In the past decade many advances have been made in tandem IM instrumentation. Transversal modulation IMS (TMIMS) [27] for example operates at atmospheric pressures, and similarly to DMA analysers, utilises electric fields. Perpendicular and oscillating fields create a stable trajectory for ions of a particular mobility. During the first publication a tandem TMIMS configuration cell was tested, which was then coupled at the front end of a mass-spectrometer, showing a resolving power of R > 80 [27, 49].

Developed later were structures for lossless ion manipulation (SLIM) [42], which formed the basis of future tandem IM devices such as the SUPER [43] and M-SLIM [44] instruments discussed later. These structures were based on TWIMS, and utilised modified RF amplitudes and DC guarding voltages in order to allow manipulation of the ion packet with almost completely negligible transmission losses. This technology could move ion packets orthogonally into ion traps before selecting species for further mobility separation. This was then explored further to create a serpentine ultralong path with extended routing (SUPER) [43]. This involved a S-shape path of SLIM transmission which could allow full or partial routing of an ion packet through a region of ∼18 m in length (Figure 2C). This could be performed multiple times allowing ion separation lengths of a 1 km, with a resolving power of R ∼ 1860 being reported. [43] In this instrument, protein conformations were found to be stable in the gas-phase for much longer periods than previously reported. This is most likely due to the specific instrument used for IM analysis, as sections of it operated at a lower pressure [69]. This was used in order to measure the mobility differences present in molecules with differential isotopic abundances.

Not discussed in this review at length, has been the application of tandem IM to aerosol analysis. Tandem DMA [51] and FAIMS instruments can often be used stand-alone coupled to detectors, such as a condensation particle counter [70] (CPC) or a Raman spectroscopy [71]. In 2018 a tandem DMA cell, modified for fragmentation of highly thermally stable volatile compounds, was coupled to a triple-quadrupole, and then faraday cup detector system [45]. These detectors allowed proof-of-concept for the fragmentation and identification of a variety of explosive compounds through the use of IM alone.

A tandem trapped ion mobility spectrometer (TIMS) has also been developed [46], which is comparable in geometry to the tandem DTIMS instruments [40, 41] discussed previously (Figure 2A). Two TIMS cells are aligned with an interface capable of gating and activation, allowing mobility selection of sub-species and ion activation. A novel aspect of TIMS is that the geometry allows an increase in the duty-cycle of fragmentation. This is through the accumulation of ions in the entrance funnel prior to single stage mobility analysis and subsequent fragmentation [72], meaning that in a tandem TIMS instrument the serial fragmentation capabilities could be greatly increased. These sorts of applications, along with the initial tandem DTIMS experiments [40, 41, 60], allow an increase in the information which can be gained from each injection, as there can be multiple rounds of separation and fragmentation at a peptide level. The implications for ‘*bottom-up*’ proteomics is that there will be increased sensitivity and resolution for samples due to the multiple rounds of separation in the instrument.

A new geometry capable of flexible tandem IM experiments was announced in 2018, and then released as part of a commercial IM–MS system: the cyclic IM QToF system from Waters Corp [47, 73–75]. The geometry presented in the cyclic instrument is unlike the linear tandem DTIMS or ion cyclotron presented previously [40, 41, 61]. The ion mobility cell is made of curved TWIMS sections, which sits orthogonally to the direction of the ion path. Ion packets can be introduced via a multi-directional array, which allows multiple modes of operation. While it may appear superficially similar to the ion cyclotron, it operates in a significantly different manner, allowing ion storage and tandem-in-time IM analysis.

Initially, the IM resolution can be scaled incrementally by increasing the number of passes an ion packet experiences in the drift-region, which was used to successfully differentiate between inverse peptides SDGRG and GRGDS after 100 passes with an R ∼ 750 [47]. However more complex analyses can be performed. The section which sits in-line with the cyclic IM cell is called the array (Figure 2D, R3), and it is flanked by both ‘*pre-array’* and ‘*post-array’* storage regions. While ion packets are traversing the drift-region, subsections of the ion packet, upon crossing the array, can be ‘*sliced’* out and stored for further analysis in the pre-array region, allowing expulsion of the remaining ions from the drift-region for mass analysis, whereby the retained ions can be analysed for IM. These *slices* can be analysed by more multi-pass or by collisional activation (CA). While multi-pass analysis has been shown to be very effective for increasing the resolution of small molecules [75], it is not as effective for larger systems such as proteins [15]. With larger systems, performing slice-CA has been shown to be far more effective for distinguishing between overlapping mobility species, as arrival time distributions for proteins appear to be more diffuse due to the inherent flexibility within these systems (Eldrid et al. [76] under review).

One of the limitations of the systems which are able to perform multi-pass analysis (cIM and SUPER-SLIM) is from ‘*roll-over*’, whereby during the multiple passes of the mobility separations, high-mobility ions overtake low-mobility ions, leading to an overlap in data and artefacts. To overcome this, multi-level SLIM [42, 43] (M-SLIM) was developed in 2020 where ions can be pushed vertically to different separation levels [44]. This cleverly circumvents collecting data over a wide mobility range without performing mobility selection (i.e. slicing in the cIMs) as higher mobility ions are elevated to undergo multiple passes separately from lower mobility species. Each of these SUPER-SLIM levels can elute ions towards mass-detection separately, preventing ion packets becoming mixed post-separation.

## What's on the horizon?

Currently there is a single commercially available tandem IM–MS instrument available, the cyclic IMS QToF from Waters [47] however Bruker is developing a tandem TIMS ToF, and the SLIMS technology is being commercialised by MOBILion [46]. The release of the cyclic IM QToF is of considerable interest to the IM–MS community, as in a single year, it has lead to an almost global doubling of the number of MS instruments capable of tandem IM, which were previously built *in-house* by highly specialised groups. We expect the field of tandem IM to increase in popularity and research output due to the expansion of the number of instruments present.

This greater accessibility of tandem IM will likely aid research in diverse fields. In particular fields which deal with highly heterogenous mixtures such as glycomics, lipidomics or petroleomics seem to be ideal targets. A recent tandem IM study on glycans showed that specific mobility species produced fragments with specific isotopic shifts, allowing identification of anomeric forms of saccharides without derivatisation [75].

DTIMS remained a standalone technique for almost 70 years before coupling with MS, and since then the field has developed to include a diverse set of separation techniques, and is commonly coupled to analytical instrumentation. Tandem IM instruments were developed and a flurry of research in the following decade has lead to significant technical advances, through creation of instruments of increased resolving power capable of measuring systems on the scale of 100s of milliseconds to seconds [15, 77]. This now brings ion mobility into the time-scale of protein folding events, something which was not available two decades ago during the nascent combination of IM and MS [58, 67, 68].

We expect the latest innovations will be incorporated into a variety of systems, allowing a variety of data collection methods to either increase the resolution of the data gathered by IM and IM–IM methods, or allow deep probing of particular subsets such as with tandem ion mobility coupled to activation of ions between successive IM rounds. It is also to be expected that these advances will be coupled with a variety of spectroscopic or fragmentation methods which will be applicable to multiple systems. There are already publications showing that a combination of IM and top-down fragmentation methods can give detailed structural information [78]. These dissociation techniques, such as infrared photodissociation (IR-PD), ultraviolet photodissociation (UV-PD) and electron capture dissociation (ECD), are proven to allow characterisation of a variety of systems to a greater degree than before [79]. Similarly the use of infrared spectroscopy has been combined with IM to give detailed conformational information on analytes [80, 81]. We can only expect the scientific output using these instruments to increase and diversify.

While the instruments developed so far allow for tandem IM experiments it is worth noting that IM can be performed to the nth level, IM^N^, experiments are also possible with some of the instruments described [15, 43, 44, 47]. Again in analogy to MS^N^ we expect that such IM^N^ experiments will also allow even further probing of structural ensembles.

As with the development of any new technology we also anticipate the development of new software. Processing and visualisation of the multi-dimensional data obtained from these experiments is complex using existing software [82].

In this review we have discussed the development and expansion of tandem IM and its' applications. The field has increased in areas of research output, applications and instrumentation developed, and very encouraging results show that it has become a highly useful analytical tool. We expect tandem ion mobility to experience the same uptake that occurred with the commercialisation of IM, which is now becoming standard addition to MS instrumentation. As with MS we expect IM to become ubiquitous in laboratories around the world.

## Perspectives

- Tandem IM coupled to mass spectrometry enables a variety of experiments to be carried out for probing in detail the conformation of ions. We have seen the most application of this technology in the biochemistry and structural biology fields. Tandem IM coupled with ion activation processes is able to give new insights on systems such as glycan structure [75] or protein conformation and unfolding [14, 76].
- Tandem IM is currently experiencing a growth of interest (Figure 1A), possibly due to the availability of instrumentation, with analytical instrumentation companies having released or are preparing to release tandem IM–MS products [46, 47].
- As the instrumentation becomes more readily available we expect to see new fragmentation techniques coupled to tandem IM. New software to analyse the multi-dimensional data will also need to be developed, as multi-dimensional data-sets are produced. Further work should ensure tandem IM–MS is made accessible to as many researchers as possible, as its potential advantages for many fields of research has not been fully realised yet.

## Open Access

Open access for this article was enabled by the participation of University College London in an all-inclusive *Read & Publish* pilot with Portland Press and the Biochemical Society under a transformative agreement with JISC.

## Abbreviations

CA: collisional activation
CCS: collision cross section
DMA: differential mobility analysis
DTIMS: drift-tube ion mobility spectrometry
FAIMS: field asymmetric waveform IMS
IM: ion mobility
IMS: ion mobility spectrometry
MS: mass spectrometry
OMS: overtone mobility spectrometry
SLIM: structures for lossless ion manipulation
TIMS: trapped IMS
TMIMS: transversal modulation IMS
TWIMS: travelling wave IMS
